# Crystal structures and other properties of ephedrone (methcathinone) hydrochloride, *N*-acetylephedrine and *N*-acetylephedrone

**DOI:** 10.1007/s11419-018-0436-7

**Published:** 2018-08-18

**Authors:** Piotr Kuś, Hubert Hellwig, Joachim Kusz, Maria Książek, Marcin Rojkiewicz, Aleksander Sochanik

**Affiliations:** 1 Department of Chemistry, University of Silesia, 9 Szkolna Street, 40-006 Katowice, Poland; 2 Institute of Physics, University of Silesia, 4 Uniwersytecka Street, 40-007 Katowice, Poland; 3 Center for Translational Research and Molecular Biology of Cancer, Maria Skłodowska-Curie Memorial Cancer Centre and Institute of Oncology, 44-100 Gliwice, Poland

**Keywords:** Methcathinone (ephedrone), Ephedrine as synthesis precursor, X-ray crystallography, Infrared spectroscopy, Raman spectroscopy, NMR spectroscopy

## Abstract

**Purpose:**

Three compounds obtained from ephedrine were identified and characterized by various instrumental analytical methods. Ephedrone (methcathinone) hydrochloride and its fundamental derivatives *N*-acetylephedrine and *N*-acetylephedrone were analyzed as precursors of a cathinone derivative.

**Methods:**

The obtained samples were analyzed by gas chromatography coupled with mass spectrometry, nuclear magnetic resonance spectroscopy, infrared and Raman spectroscopy, and X-ray crystallography.

**Results:**

The three compounds were confirmed as: *N*-methyl-2-amino-1-phenylpropan-1-one (methcathinone) hydrochloride, *N*-acetyl-*N*-methyl-2-amino-1-phenylpropan-1-one (cathinone derivative), and *N*-acetyl-*N*-methyl-2-amino-1-phenylpropan-1-ol (acetyl derivative of ephedrine).

**Conclusions:**

X-ray crystallography is especially useful for identifying the new designer drugs and their different precursor forms.

**Electronic supplementary material:**

The online version of this article (10.1007/s11419-018-0436-7) contains supplementary material, which is available to authorized users.

## Introduction

**Fig. 1:**
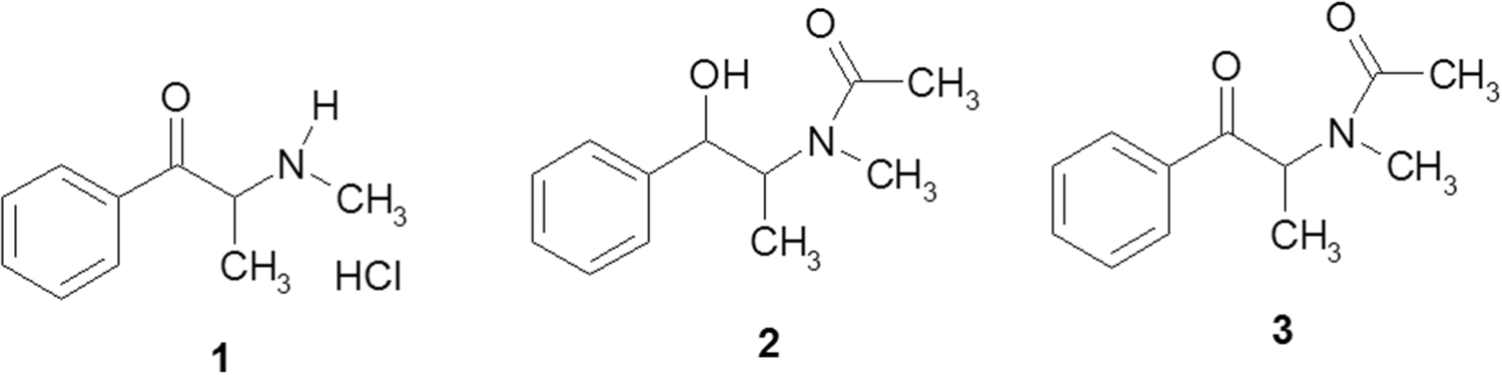
Structures of *N*-methyl-2-amino-1-phenylpropan-1-one (ephedrone) hydrochloride (**1**); *N*-acetylephedrine (**2**), and *N*-acetyl-*N*-methyl-2-amino-1-phenylpropan-1-one (*N*-acetylephedrone) (**3**)

Ephedrone (**1**) is one of the oldest known synthetic cathinones [1–3]. Its production has been based on ephedrine [(*1R*,*2S*)-1-phenyl-1-hydroxy-2-(*N*-methylamino)propane], a substance occurring naturally in shrubs of the genus *Ephedra*, native to parts of Europe, Asia, and the Americas. The ephedrine molecule contains two chiral carbon atoms and can therefore occur as four chiral isomers: two *erythro* [l-(−)-ephedrine and d-(+)-ephedrine], and two *threo* isomers [(−)-pseudoephedrine and (+)-pseudoephedrine] (Fig. 1). Levorotatory ephedrine occurs naturally.

Ephedrine (in general) has been listed as a drug precursor in Regulation No. 273/2004 of the European Parliament and of the Council (Annex 1).

Numerous anti-inflammatory, antipyretic, and analgesic drugs available over the counter contain pseudoephedrine, from which ephedrone can be produced at home via oxidation of the partially separated component of the medication. Pseudoephedrine contained in pills is oxidized using potassium permanganate and an acetic acid milieu [4, 5] or, alternatively, using potassium dichromate in sulfuric acid [6]. Procedures of this type make it impossible to completely remove manganese or chromium ions from the mixture; therefore, compounds containing these elements can enter the human body. Literature data include reports linking the presence of manganese ions delivered in such a manner with Parkinson’s disease, possibly as a causative factor [7, 8]. Organic synthetic procedures for obtaining ephedrone from ephedrine and its analogues have also been reported [9–11].

Data concerning the crystallographic structures of ephedrone derivatives have been reported for metaphedrone hydrochloride [12], 2-MMC and 4-CMC hydrochlorides [13], methylone, mephedrone, and 1-(3,4-dimethylphenyl)-2-(methylamino)propan-1-one HCl [14]. For mephedrone derivatives, conditions have been specified (change of halide ion) that alter its physicochemical characteristics (phase transition temperatures and melting points) [15].

Despite no reported use of acetylated ephedrone derivative **3 **as a designer drug, we determined the spectroscopic and crystallographic properties of this cathinone derivative, as well as those of its precursor, compound **2**, which is an acetylated ephedrine. The apparent lack of interest in this compound as a designer drug may result from its poor solubility in water.

## Materials and methods

Deuterated dimethyl sulfoxide (DMSO-*d*_*6*_), deuterated chloroform (CDCl_3_) and other chemicals were purchased from Sigma-Aldrich (Poznań, Poland). Melting points were uncorrected.

The nuclear magnetic resonance (NMR) spectra were recorded using an UltraShield 400 MHz apparatus (Bruker, Bremen, Germany) with CDCl_3_ or DMSO-*d*_*6*_ as solvent. The peaks were referenced to the residual chloroform (CHCl_3_; 7.28 and 77.04 ppm) and dimethyl sulfoxide (DMSO; 2.49 and 39.5 ppm) resonances in ^1^H and ^13^C NMR. The NMR data are presented in the Supplementary Material.

The infrared (IR) spectra of each compound were obtained using a Nicolet iS50 Fourier transform infrared (FTIR) spectrometer (Thermo Scientific, Warsaw, Poland) and the attenuated total reflectance technique. Raman measurements were performed using a Thermo Scientific™ DXR™2xi Raman imaging microscope equipped with a 780-nm laser (Thermo Scientific).

Gas chromatography–mass spectrometry (GC–MS) analyses were performed using a Thermo Trace GC Ultra chromatograph coupled to a mass spectrometer (Thermo DSQ; Thermo Scientific). The injector was maintained at 260 °C. A 1-μL aliquot of the sample was injected in the splitless mode. Separation of sample components was conducted using the Rxi^®^-5Sil MS column (30 m length, 0.25 mm inner diameter, 0.25 µm film thickness; Restek, Bellefonte, PA, USA). Helium was used as carrier gas at a flow rate of 1.2 mL/min. The mass detector was set to positive electron ionization (EI) mode, with electron energy of 70 eV. The mass detector was operated in a full-scan mode in the 40–450-amu range.

The single-crystal X-ray experiments were performed at 100 K for compounds **1** and **3**, and at 293 K for compound **2**. The data were collected using a SuperNova kappa diffractometer with an Atlas charge-coupled device detector (Rigaku Europe, Chalgrove, UK). For the integration of the collected data, CrysAlis^Pro^ software (version 1.171.38.41q, 2015; Rigaku Europe) was used. The solving and refining procedures were similar for all compounds. The structures were solved using direct methods with SHELXS97 software, and the solutions were refined using SHELXL-2014/7 software [16]. CCDC 1816306 for **1**, CCDC1819495 for** 2**, and CCDC 1816307 for **3** are included in the supplementary crystallographic data as part of this paper. These data can be obtained free of charge from the Cambridge Crystallographic Data Centre ( www.ccdc.cam.ac.uk/data_request/cif).

## Results and discussion

**Fig. 2:**
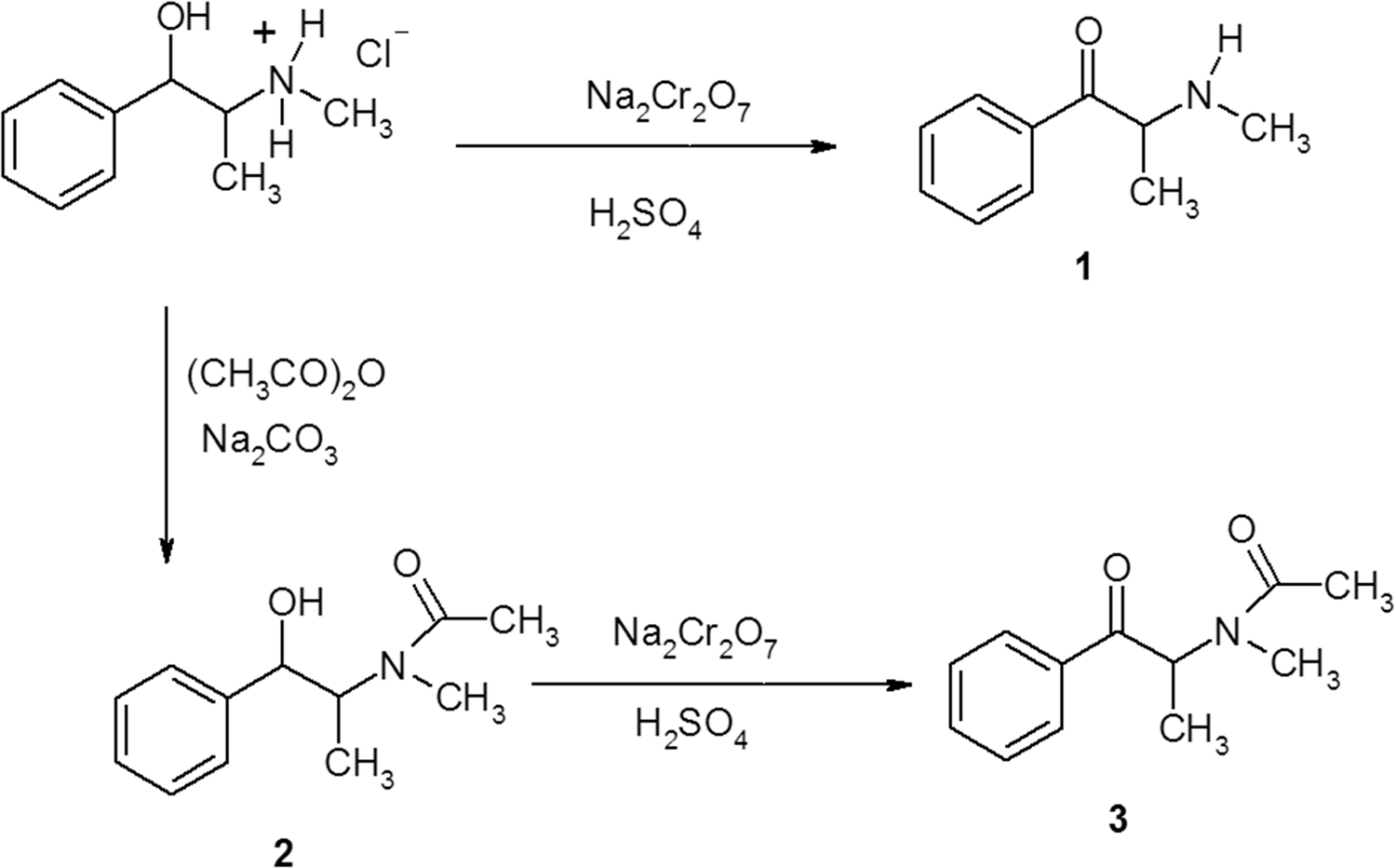
Preparation route for compounds **1**–**3** from l-(−)-ephedrine hydrochloride

Compounds **1**–**3** required for X-ray analysis were obtained from l-(−)-ephedrine hydrochloride at 5 mmol reaction scale, according to the scheme shown in Fig. 2. Compound **1** was synthesized from ephedrine according to the procedure described in [10] and compared with the materials seized by drug enforcement agencies on the illicit drug market. Compound **2** was obtained according to the procedure reported in [17]. It occurs as a mixture of two rotamers (1.1:1 ratio), as described previously (a 2.8:1 rotamer mixture) [18]. Oxidation of compound **2** was carried out in analogy with obtaining compound **1**. Particular intermediate products were obtained with moderate yield (40%). Reaction efficiencies were not optimized. Physicochemical data pertaining to compounds **1** and **2** were identical with those reported (although compound **2** was obtained in [18] as a colorless liquid). Compound **3** was obtained in [17] as a colorless liquid (no information was given concerning isomer type). We obtained all three compounds as solids, and their structures were corroborated by the crystallographic study.

### GC–MS spectra

Chromatography analyses of ephedrone have been frequently reported [19, 20]. The GC–MS spectra of this generated compound in our study were compared with those of the other two. These data can be helpful in analyzing other possible ephedrine derivatives. Signals from molecular ions of all three compounds were present at low intensities, at 5–15%. Iminium ions (*m/z* = 58) were observed to be dominant for all compounds. In the case of ephedrone (**1**), it is formed in a single process of bond cleavage between carbon atoms α and β in the side chain. In the case of compounds **2** and **3**, beside the cleavage just described above, there was another cleavage present (that of an acetyl group from the nitrogen atom). The mass spectral fragmentation patterns of all compounds are presented in the Supplementary Material (Figs. S1 and S2).

### NMR spectra

The structure of compound **1** has been corroborated by ^1^H NMR and ^13^C NMR spectra. ^1^H NMR data for this compound were published previously but for another solvent system (DMSO-*d*_6_ + D_2_O) [11]. For this particular solvent mixture, the authors observed a multiplet of benzene ring protons. When using pure DMSO-*d*_6_ as a solvent, particular protons were distinguished as a doublet at *δ* = 8.04 ppm (*J *= 8 Hz) from *ortho* protons, a triplet at *δ* = 7.76 ppm (*J *= 8 Hz) from *para* protons and a triplet at *δ* = 7.62 ppm (*J *= 8 Hz) from *meta* protons in the benzene ring (see Supplementary Material). A similar spectrum was also reported in [9], although it is difficult to say whether it represented pure ephedrone or ephedrone hydrochloride; the lack of any signal from amine proton and good solubility in chloroform would suggest the presence of the former species. Another paper [21] reported two spectra identical to ours, which also had no NH proton signals. These spectra were obtained using CDCl_3_ and CD_3_OD. In our spectra, a broad signal of amine protons at δ ~ 9.25 ppm was clearly distinguishable. ^13^C NMR spectra were given in [9] and [21]. Concerning [9], the authors provided signal shift values for all carbon atoms in the molecule, including that for carbonyl carbon, which in itself was proof of obtaining ephedrone from ephedrine. In [21], the signal from this carbon atom was clearly seen at *δ* = 195.22 ppm. In our study, this shift was *δ* = 196.8 ppm. The remaining signals are presented in the Supplementary Material.

Compound **2** occurred as two rotamers at an almost equimolar ratio (1.1:1 value derived from NMR spectrum). Particular protons and carbons from both rotamers displayed various signal shifts in ^1^H NMR and ^13^C NMR spectra, respectively. Aromatic protons in compound **2** occurred as a complex multiplet. Proton signals from the OH groups of both rotamers occurred adjacent to one another; the signals were stretched out, and their position was sample concentration-dependent, as well as dependent on the presence of other molecules (e.g., water) in the solvent. The proton signals occurred at 5.43 and 5.65 ppm (see Supplementary Material). All the remaining protons occurred as two distinct signals, the ascription of which to a particular rotamer type can be determined by two-dimensional correlation spectroscopy. In ^13^C NMR spectra, the signals of all carbon atoms from rotamer molecules occurred as double peaks. Such spectra contained only one (double) signal from the carbonyl group at *δ* = 170.0 ppm (169.7 ppm for the rotamer) in the acetyl moiety. Likewise, the signal from carbon in C–OH occurred only at *δ* = 75.1 ppm (74.8 ppm for the rotamer), which showed that no acylation of the hydroxyl group took place in compound **2**; the possibility of obtaining an acetyl derivative was reported in [22, 23]. The remaining signals are presented in the Supplementary Material. The first ^1^H NMR spectra of compound **2** published in [17] and [24] did not show the presence of double signals for particular proton groups; it was suggested that this occurs only in the case of the compound’s enantiomer mixture. A subsequent report [25] confirmed the occurrence of rotamers in pure enantiomers of compound **2** and doubling of ^1^H NMR spectrum signals.

Compound **2** in solution can theoretically occur in three conformations, which are shown in Fig. S3 (Supplementary Material). As is obvious from crystallographic studies described below, the presence of conformer **2a** in the crystal has been confirmed. Based on this knowledge and on ^1^H NMR spectral data, the other conformer present in solutions of this compound  was **2c**. This is in accordance with earlier conformational [26] and spectroscopic [27] analyses of ephedrine.

Compound **3** was characterized based on well-separated peaks from aromatic protons: a doublet at *δ* = 7.96 ppm (*J *= 8 Hz) from *ortho* protons, a triplet at *δ* = 7.55 ppm (*J *= 8 Hz) from *para* proton, and a triplet at *δ* = 7.44 ppm (*J *= 8 Hz) from *meta* protons of the benzene ring. The lack of signal from amine group protons confirmed its acylation. The peak of C–H proton was strongly shifted (*δ* = 6.15 ppm). The ^13^C NMR spectrum was characterized by the presence of signals from two carbonyl carbon atoms: one presented in an amide group (*δ* = 170.4 ppm) and the other in Ar–CO (*δ* = 199.3 ppm). The remaining peaks occurred in the expected positions.

### IR and Raman spectra

IR and Raman spectra can be useful for characterizing the synthesis of designer drugs. Spectral analysis of mixtures can reveal much information about particular stages of synthesis. In the case of ephedrone and its derivatives synthesized from ephedrine, pseudoephedrine or their derivatives (e.g., acetylated derivatives), characteristic vibrations of ketone or hydroxyl groups allowed the assessment of a putative route of synthesis for an examined compound. FTIR and Raman FT spectra of compound **1** were reported in [21]. These spectra are identical with those (denoted as Figs. S4 and S7) generated in our study, and presented together with spectra of compounds **2** (Figs. S5 and S8) and **3** (Figs. S6 and S9), respectively (see Supplementary Material).

Vibrations of the carbonyl group from a propionic fragment occurred in similar positions in the FTIR spectra of compounds **1** and **3** (1687 and 1688 cm^−1^, respectively). Compound **3**  had yet another carbonyl group vibrational band at 1629 cm^−1^, originating from this compound’s acetyl moiety. The spectrum of compound **2** featured one strong vibrational CO band from an acetyl fragment at 1602 cm^−1^, as well as a very strong band at 3275 cm^−1^, characteristic of vibrations of OH.

In the Raman spectra of compounds **1**–**3**, the most characteristic were C=O and aromatic ring vibrations. The C=O absorptions appeared at 1686 cm^−1^ for **1**, at 1603 cm^−1^ for **2**, and at 1686 cm^−1^ and 1628 cm^−1^ for **3**. The aromatic ring vibrations appeared at 1597, 1580, and 1596 cm^−1^ for compounds **1**–**3**, respectively.

### X-ray analysis

**Fig. 3:**
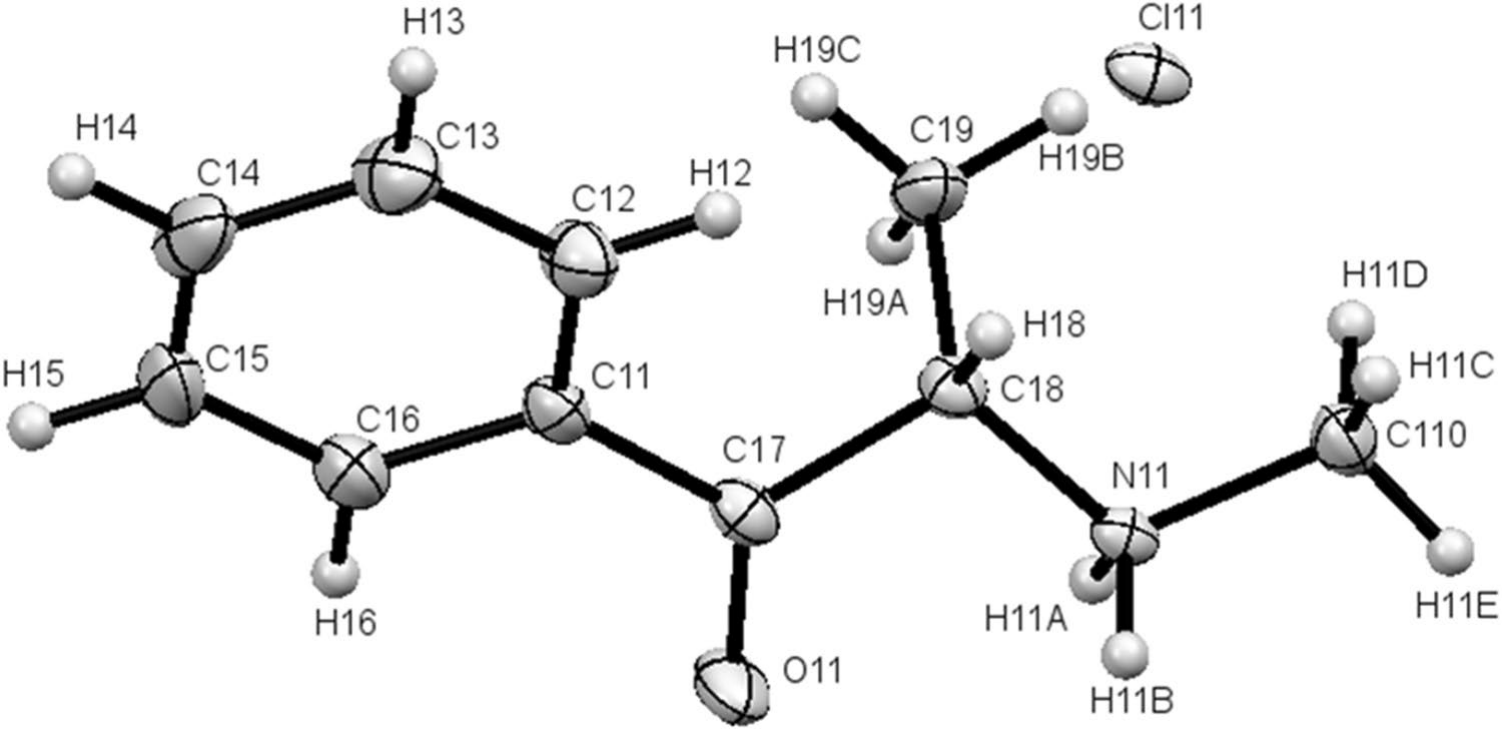
(*S*)-Enantiomer molecule of compound **1** in the crystal. Ellipsoids for non-H atoms correspond to 50% probability levels

The salt **1** was crystallized from DMSO solution as colorless plate-like crystals. Compound **2** was crystallized from water, also as colorless plate-like crystals. Compound **3** was crystallized from chloroform solution as colorless needle-shaped crystals. The crystal structures of compound **1** are shown in Fig. 3 (crystal structures for **2** and **3** are shown in Figs. S10 and S11, whereas crystal packing is shown in Figs. S14 and S15, respectively). Crystal data and structure refinement for compounds **1**–**3** are presented in Table 1.

**Table 1:** Crystal data, data collection, and structure refinement for compounds **1**–**3**

	**1**	**2**	**3**
Chemical formula	C_10_H_14_N_1_O_1_Cl_1_	C_12_H_17_N_1_O_2_	C_12_H_15_N_1_O_2_
Molecular weight	199.67	207.26	205.25
Temperature (K)	100	293	100
Wavelength (Å)	0.71073	0.71073	0.71073
Crystal system	Orthorhombic	Tetragonal	Orthorhombic
Space group	*P 2*_*1*_ *2*_*1*_ *2*_*1*_	*P 4*_*3*_ *2*_*1*_ *2*_1_	*P 2*_*1*_ *2*_*1*_ *2*_*1*_
Unit cell dimensions			
*a* (Å)	10.0074(2)	7.1249(1)	9.0265(1)
*b* (Å)	10.1833(1)	7.1249(1)	10.0699(2)
*c* (Å)	44.3015(8)	44.1371(15)	12.3493(2)
*β* (°)	90	90	90
* V* (Å^3^)	4514.69(13)	2240.58(10)	1122.50(3)
*D*_calc_ (mg m^−3^)	1.175	1.229	1.215
*Z*	16	8	4
Absorption coefficient (mm^−1^)	0.303	0.083	0.083
*F*(000)	1696	896	440
Crystal size (mm^3^)	0.12 × 0.10 × 0.07	0.12 × 0.10 × 0.10	0.09 × 0.08 × 0.02
Theta range for data collection (°)	2.891–26.373	3.004–36.342	3.031–26.368
Index ranges	− 12 ≤ h ≤ 12	− 11 ≤ h ≤ 11	− 10 ≤ h ≤ 11
	− 12 ≤ k ≤ 12	− 11 ≤ k ≤ 11	− 11 ≤ k ≤ 12
	− 55 ≤ l ≤ 55	− 67 ≤ l ≤ 70	− 15 ≤ l ≤ 15
Reflections collected	46577	41278	9410
Independent reflections	9220 [*R*_(int)_ = 0.0346]	5245[*R*_(int)_ = 0.0893]	2290[*R*_(int)_ = 0.0186]
Data/restraints/parameters	9220/0/478	5245/0/141	2290/0/139
Goodness of fit on *F*^2^	1.135	0.984	1.044
Final *R* indices [*I *> 2*σ*(*I*)]	*R*1 = 0.0349	*R*1 = 0.0604	*R*1 = 0.0250
	*wR*2 = 0.0748	*wR*2 = 0.1187	*wR*2 = 0.0671
*R* indices (all data)	*R*1 = 0.0367	*R*1 = 0.1559	*R*1 = 0.0256
	*wR*2 = 0.0756	*wR*2 = 0.1500	*wR*2 = 0.0676
Largest diff. peak and hole (e Å^−3^)	0.288 and − 0.306	0.167 and − 0.177	0.158 and − 0.131

**Fig. 4:**
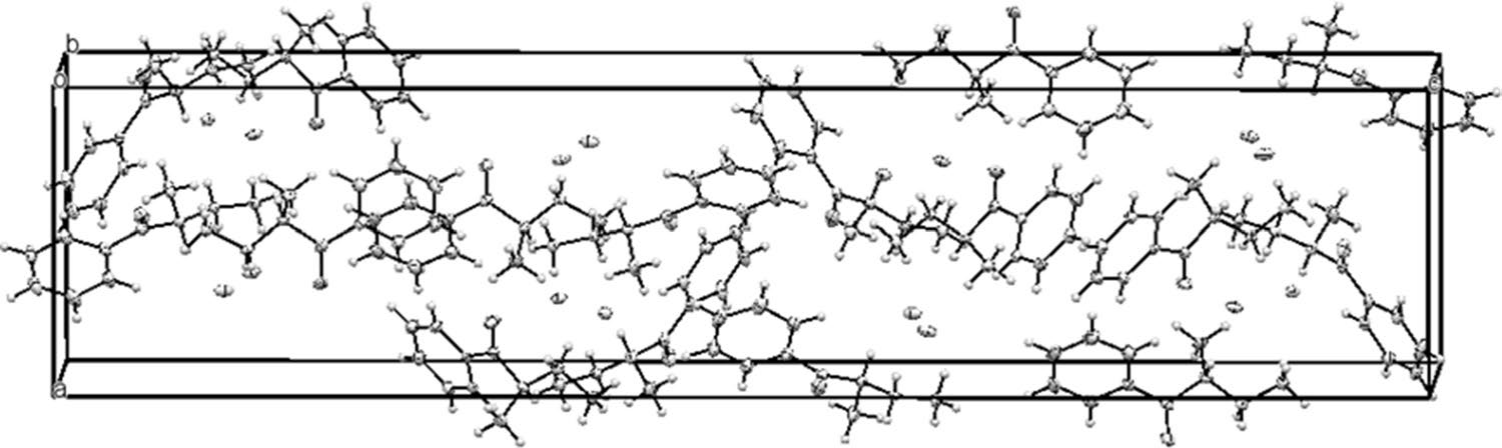
Packing diagram for **1**: view along the *b* axis

Both cathinones **1** and **3** occurred as expected *S*-isomers, which was in accordance with the mechanism of their formation from l-(−)-ephedrine. Both molecules crystallized in orthorhombic space groups. In the crystallographic structure of compound **1**, an important role was assigned to the N–H···Cl^−^ hydrogen bonds. The N–H donors (from R_1_R_2_NH_2_^+^ groups) donated the hydrogen bonds to chloride anions separated from one another by a distance of 2.166 to 2.280 Å, with N–H···Cl^−^ angles from 147.89° to 157.32°. The separation of N···Cl^−^ was between 3.017 and 3.089 Å. Distances and angles listed above were similar to those reported in [28]. Analysis of crystal packing in compound **1** revealed much empty space between molecules, especially considering the packing along the *b* axis of the elementary cell (Fig. 4). The schemes of crystal packing along other axes are presented in Figs. S12 and S13. Crystal packing for compounds **2** and **3** is shown only along the *b* axis in Figs. S14 and S15, respectively.

*N*-Acetylephedrine **(2)** crystal which we managed to obtain from aqueous solution contained  only one kind of conformer in the elementary cell. No other conformer was found to be present in solution, and this was corroborated by NMR spectra, both in this study and in [24, 25]. The hydroxyl group in compound **2** played  a fundamental role in the arrangement of particular molecular fragments relative to one another, which is likely of importance to the packing of molecules in the elementary cell. The presence of an intramolecular C9–H···O1 hydrogen bond of 2.503 Å could be noted, although the D–H···A angle  was only 123.39°, along with an intermolecular hydrogen bond between the OH group of one molecule and the oxygen atom from an adjacent acetyl group (length 1.989 Å, angle D–H···A 171.15°). The positioning of the phenyl ring with respect to the molecule’s skeleton may be stabilized by a weak hydrogen bond C_Ar_–H···OH (length 2.625 Å, angle D–H···A—140.56°), as well as with a C–H···Ar_centr_ bond (length 2.684 Å, angle D–H···A—143.88°). The characterized crystal of compound **2** featured  conformer **2a** (Fig. S17). No other stable conformers could be confirmed in the examined crystal.

Compound **2**’s conformation was “forced” by the occurrence of weak hydrogen bonds between oxygen atoms (O1 and O2) and hydrogen atoms from methyl groups at the C9 nitrogen and C12 carbon. All distances which could be determined between hydrogen atoms and their acceptors (oxygen atoms)  were shorter (2.246, 2.503 and 2.551 Å) than the sum of the van der Waals radii for these elements (2.72 Å) (Fig. S18).

The crystallographic structure of compound **3** differed significantly from that of compound **2**. Due to the lack of an ionized fragment in the molecule, which would facilitate its solubility in water, it also lacked  strong intermolecular hydrogen bonds. The acetamide fragment  was planar with an almost ideal Nsp^2^ atom. This fragment existed in the mesomeric form characteristic for peptide bonds, in which a single bond occurs between C and O atoms and a double bond between C and N atoms.

Weak hydrogen bonds occurred between oxygen atoms from carbonyl groups (both at C7 atom and in acetyl substituent; Fig. S16 in Supplementary Material) and protons from adjacent molecules. One can also find a few weak intramolecular hydrogen bonds which stabilize a characteristic bend of the benzene ring with respect to the plane of the amide fragment (see Supplementary Material).

## Conclusions

Ephedrone, which is easily made at home from legally available drugs, may be among the most frequently abused designer narcotics. Methods of obtaining this harmful compound are available on the web. Despite straightforward synthesis, the end product is contaminated with manganese ions, believed to be involved in the development of drug-induced parkinsonism. Spectroscopic and crystallographic data concerning ephedrone and its acetylated derivatives could be helpful in assessing evidence in criminal proceedings. The NMR spectra of compound **2** suggest the existence of two stable conformers of this compound in solution, although the crystallographic structure determined during analysis of aqueous solution-derived crystals demonstrated the presence of only one type of conformer.

## Electronic supplementary material

Below is the link to the electronic supplementary material.

Supplementary material 1 (DOC 1197 kb)
