# Supplementary material for: Crystal structures and other properties of ephedrone (methcathinone) hydrochloride, N-acetylephedrine and N-acetylephedrone
Source: Forensic Toxicol. 2018 Aug 18;37(1):224–30. doi: 10.1007/s11419-018-0436-7 (PMC6315050; doi:10.1007/s11419-018-0436-7)
Supplement: Supplementary file 1 — Supplementary material 1 (DOC 1197 kb) [file 11419_2018_436_MOESM1_ESM.doc]

Supplementary material

**Crystal structures and other properties of ephedrone (methcathinone) hydrochloride, *N*-acetylephedrine and *N*-acetylephedrone**

Piotr Kuś^1^, Hubert Hellwig^1^, Joachim Kusz^2^, Maria Książek^2^, Marcin Rojkiewicz^1^, Aleksander Sochanik^3^

**Melting points and NMR data of compounds**  **1** – **3**

*(S) – Ephedrone hydrochloride* (**1**). M.p. 176 – 179 ^o^C (dec.) (lit. 176 – 179 ^o^C [11]).

^1^H NMR (DMSO-*d_6_*) δ (ppm): 9.25 (bs, 2H, NH), 8.04 (d, 2H, *J* = 8 Hz, *o*-Ar), 7.76 (t, 1H, *J* = 8 Hz, *p*-Ar), 7.62 (t, 2H, *J* = 8 Hz, *m*-Ar), 5.15 (q, 1H, C-H), 2.61 (s, 3H, N-CH_3_), 1.45 (d, 3H, C-CH_3_).

^13^C NMR (DMSO-*d_6_*) δ (ppm): 196.8, 135.1, 133.5, 129.6, 129.2, 58.7, 31.1, 15.8.

*(1R,2S)-N-Acetylephedrine* (**2**). (1.1:1* rotamer ratio; asterisk denotes second rotamer peak). M.p. 80 – 86 ^o^C (lit. 87 – 88 ^o^C [17]; 85 – 86 ^o^C [18]; colorless oil in [19]).

^1^H NMR (DMSO-*d_6_*) δ (ppm): 7.20-7.35 (m, 10H, ArH + ArH*), 5.65 (bs, 1H, OH), 5.43 (bs, 1H, OH*), 4.59 (d, 1H, C_OH_-H), 4.52 (d, 1H, C_OH_-H*), 4.52 (q, 1H, C-H), 3.83 (q, 1H, C-H*), 2.81 (s, 3H, N-CH_3_), 2.66 (s, 3H, N-CH_3_*), 1.81 (s, 3H, CO-CH_3_), 1.62 (s, 3H, CO-CH_3_*), 1.26 (d, 3H, C-CH_3_), 1.06 (d, 3H, C-CH_3_*).

^13^C NMR (DMSO-*d_6_*,) δ (ppm): 170.0, 169.7*, 144.2, 144.1*, 128.3*, 128.2, 127.7*, 127.3, 126.8*, 126.6, 75.1, 74.8*, 59.2, 54.3*, 32.0*, 28.0, 22.55, 21.7*, 15.29, 12.6*.

*(S)-N-Acetylephedrone* (**3**). M.p. 76 – 77 ^o^C (lit. colorless oil in [17]).

^1^H NMR (CDCl_3_) δ (ppm): 7.96 (d, 2H, *J* = 8 Hz, *o*-Ar), 7.55 (t, 1H, *J* = 8 Hz, *p*-Ar), 7.44 (t, 2H, *J* = 8 Hz, *m*-Ar), 6.15 (q, 1H), 2.79 (s, 3H, N-CH_3_), 2.07 (s, 3H, CO-CH_3_), 1.35 (d, 3H, C-CH_3_)

^13^C NMR (CDCl_3_) δ (ppm): 199.3, 170.4, 135.2, 133.4, 128.7, 128.4, 52.7, 31.3, 21.9, 13.2.


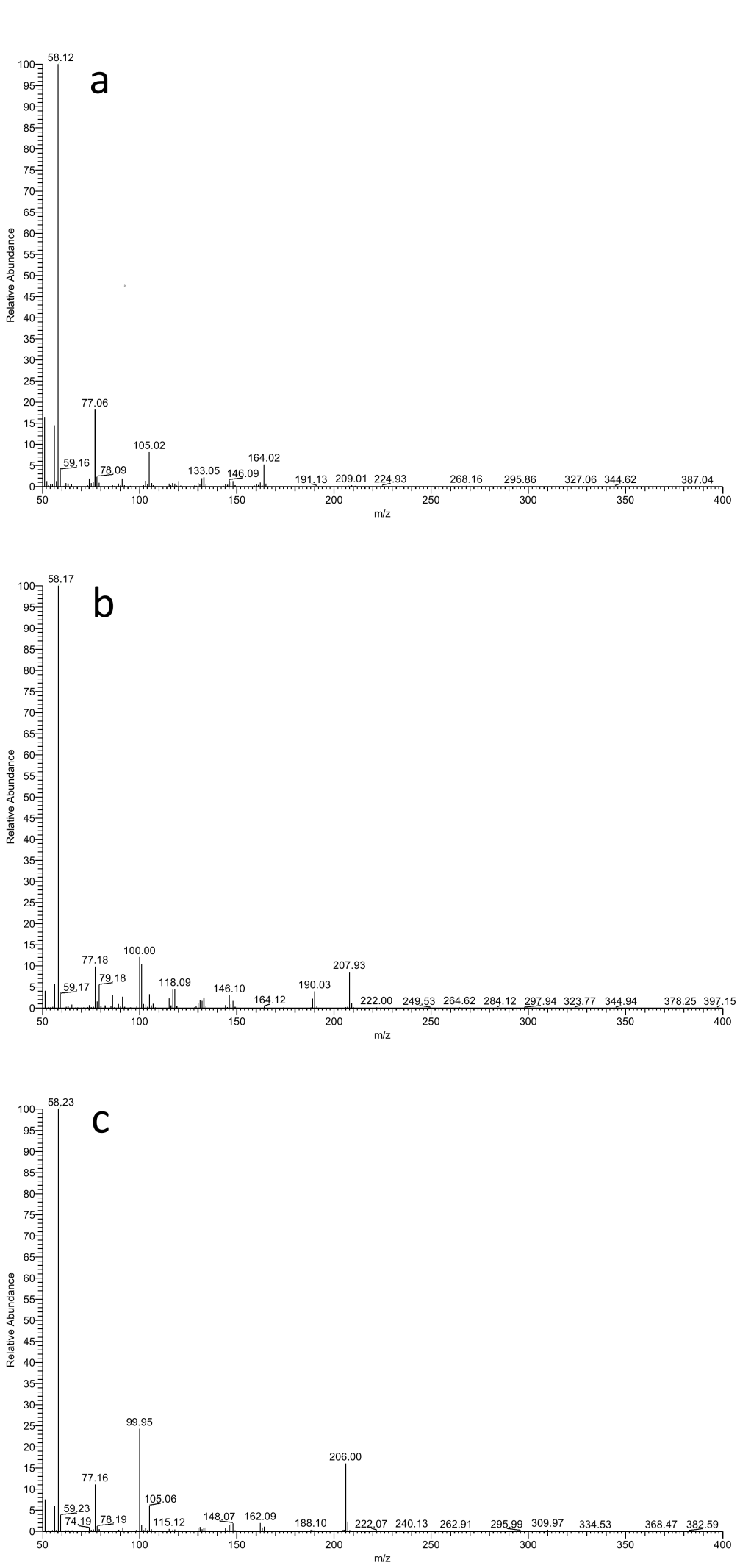


**Fig. S1** Electron ionization mass spectra of compounds: **1** (**a**), **2** (**b**) and **3** (**c**)

**a**

**b**

**c**

**Fig. S2** Mass-spectral fragmentation patterns of: **a** ephedrone (**1**); **b** *N*-acetylephedrine (**2**) and **c** *N*-acetylephedrone (**3**)

**Fig. S3** Newman projections of possible conformers of compound **2**


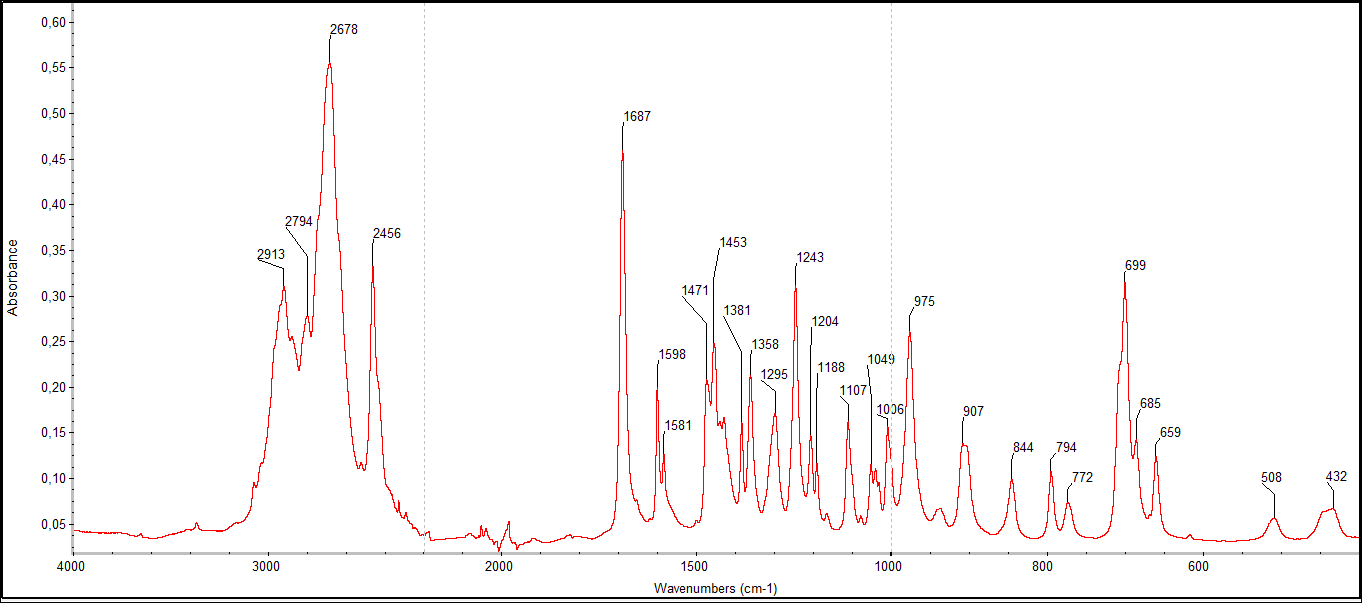


**Fig. S4** FTIR spectrum of compound **1**


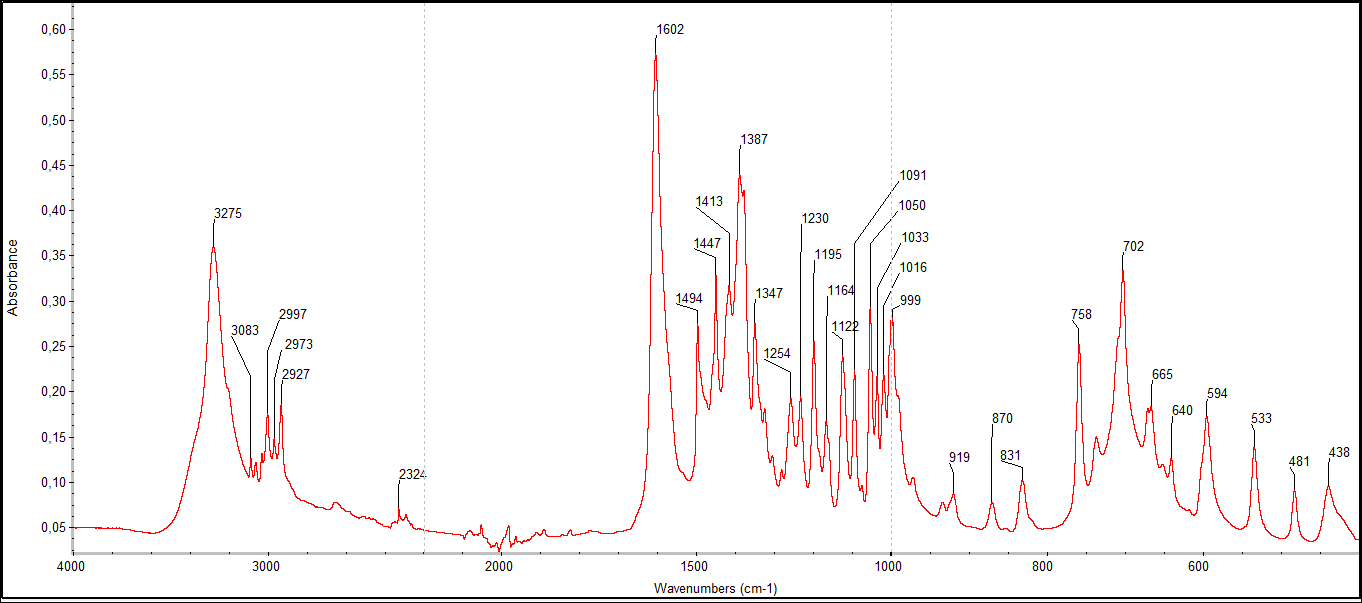


**Fig. S5** FTIR spectrum of compound **2**


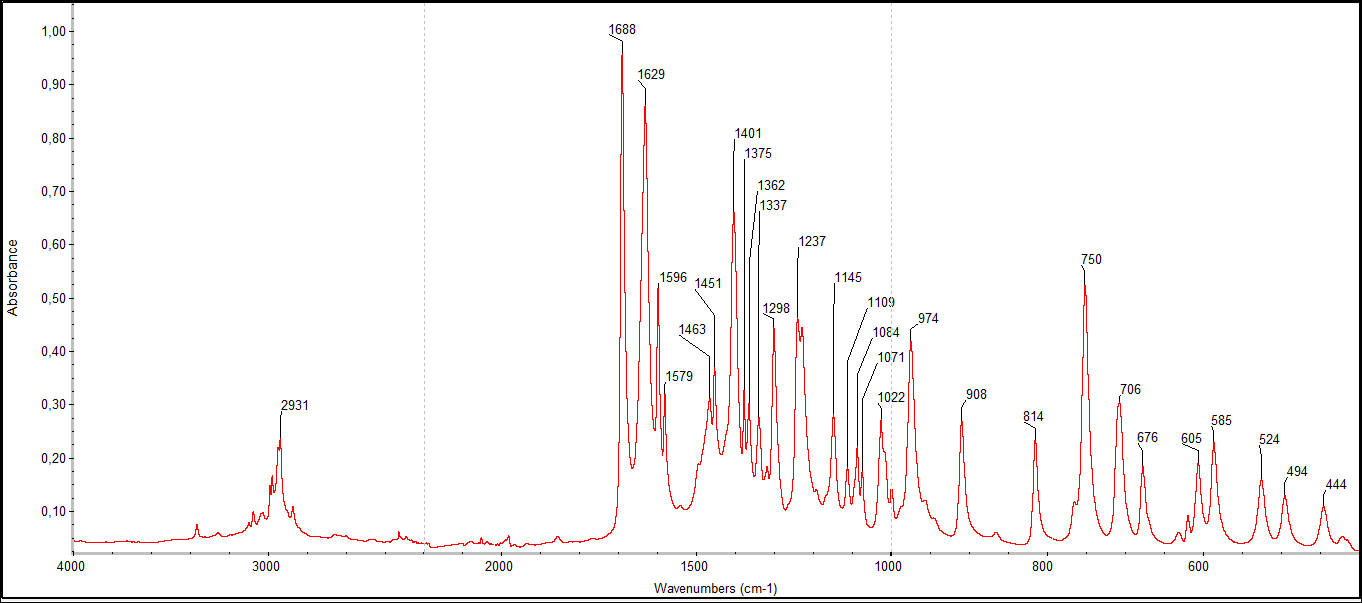


**Fig. S6** FTIR spectrum of compound **3**

**
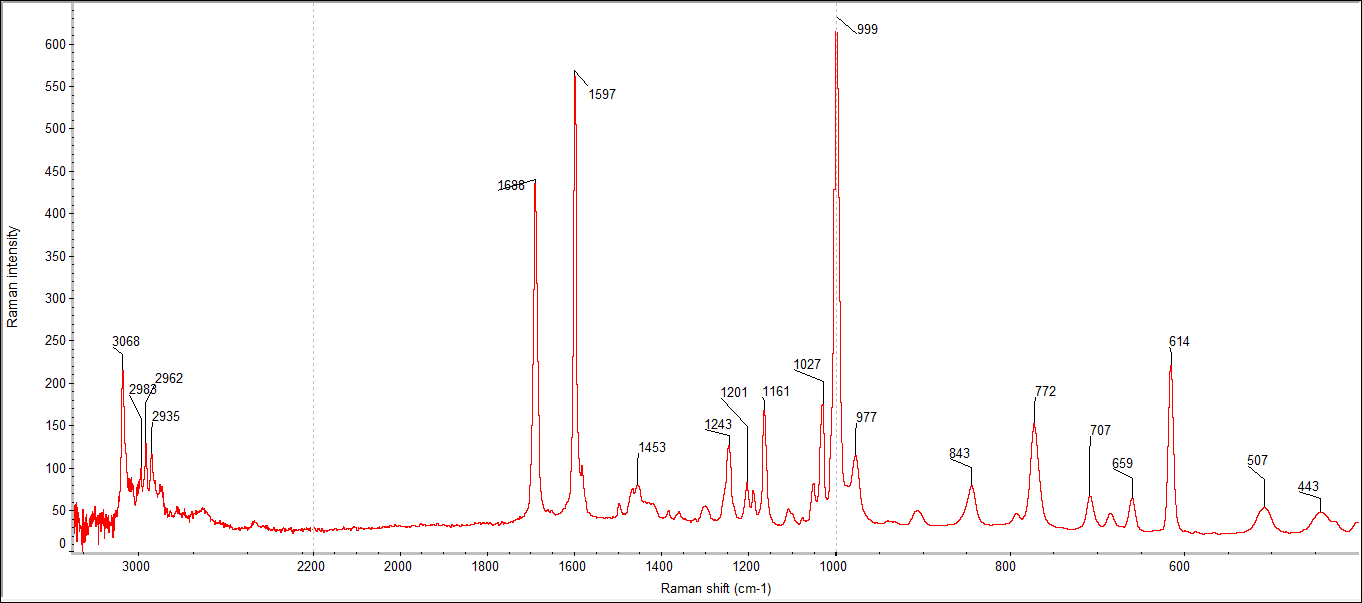
**

**Fig. S7** Raman spectrum of compound **1**


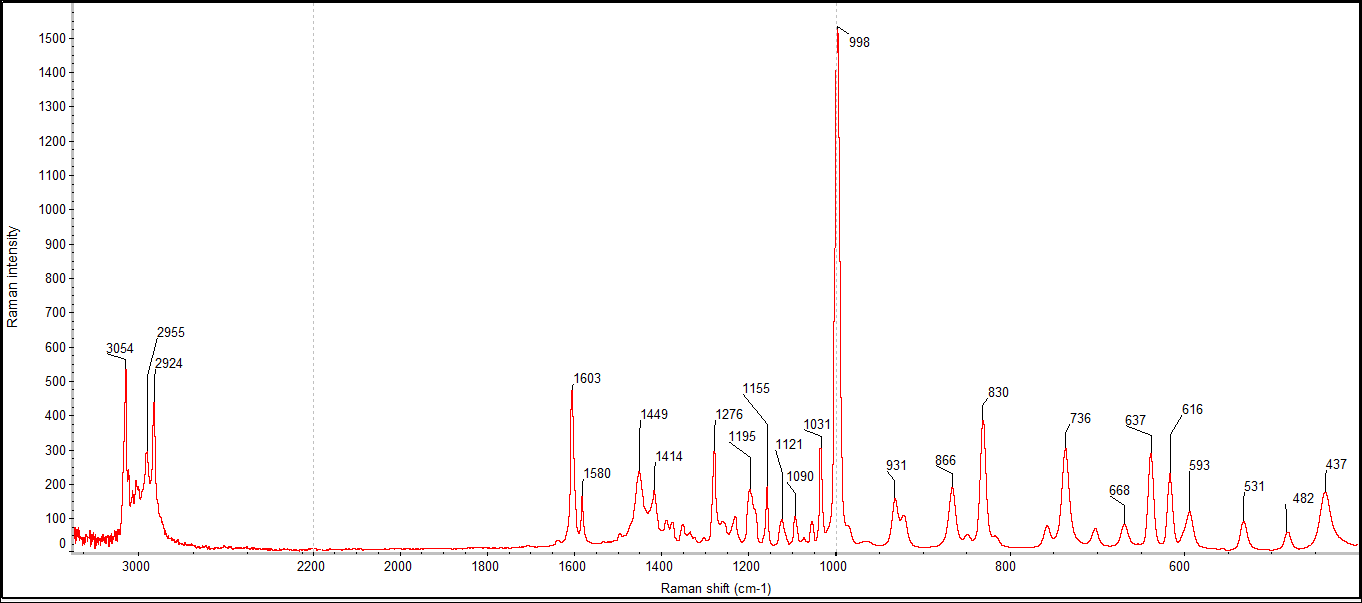


**Fig. S8** Raman spectrum of compound **2**


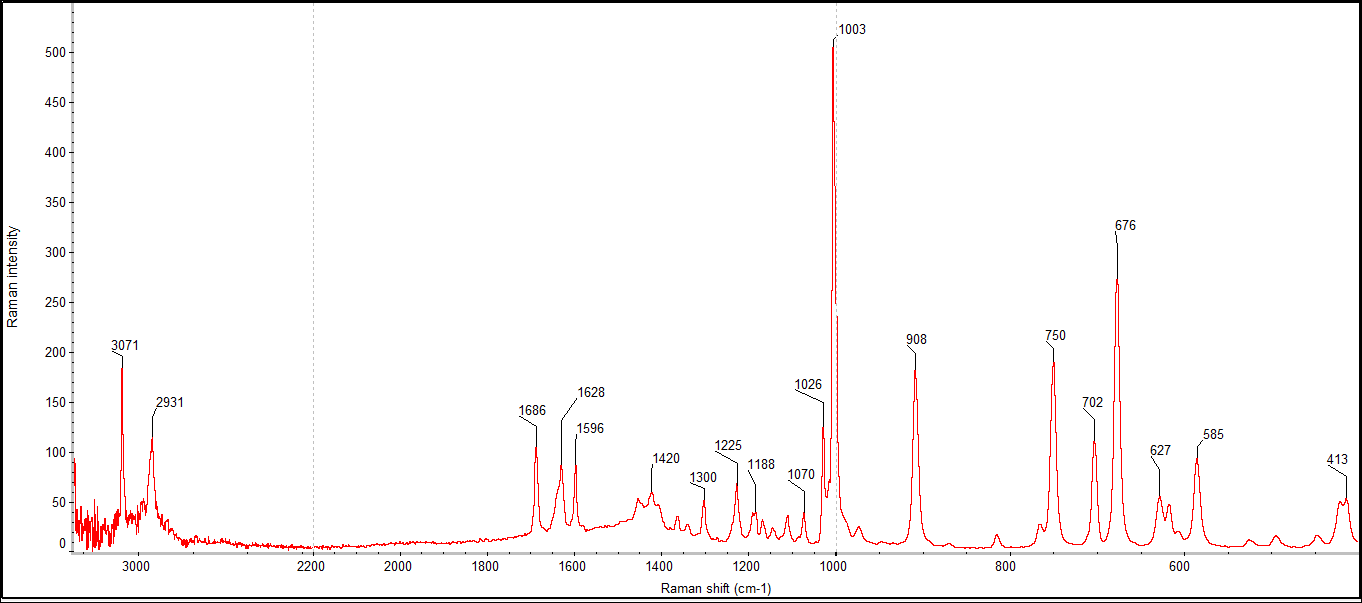


**Fig. S9** Raman spectrum of compound **3**


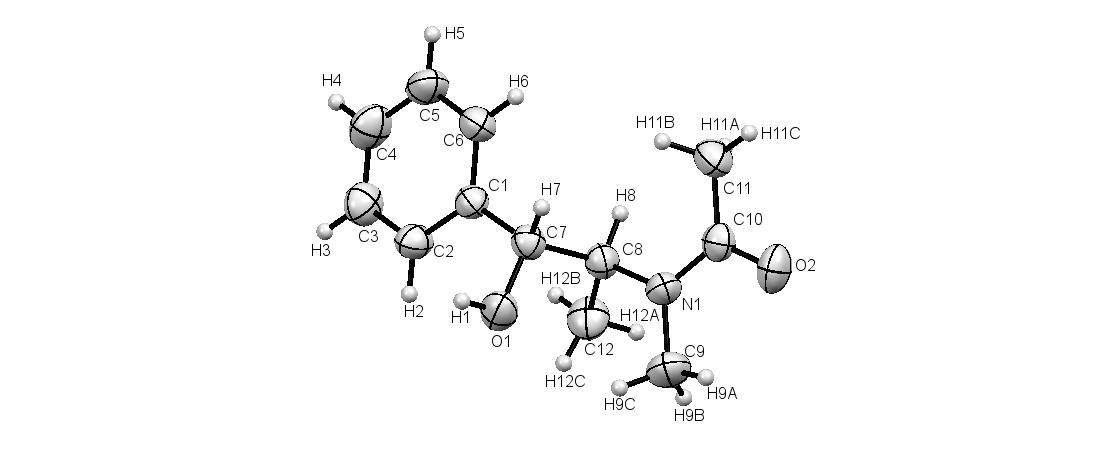


**Fig. S10** The molecule of compound **2** in the crystal. Ellipsoids correspond to 50% probability levels


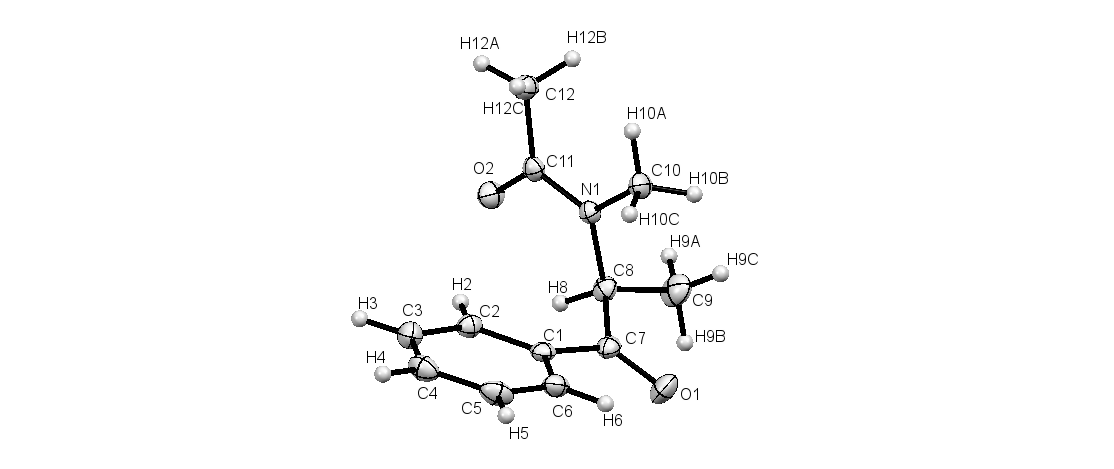


**Fig. S11** (S)-Enantiomer molecule of compound **3** in the crystal. Ellipsoids correspond to 50% probability levels


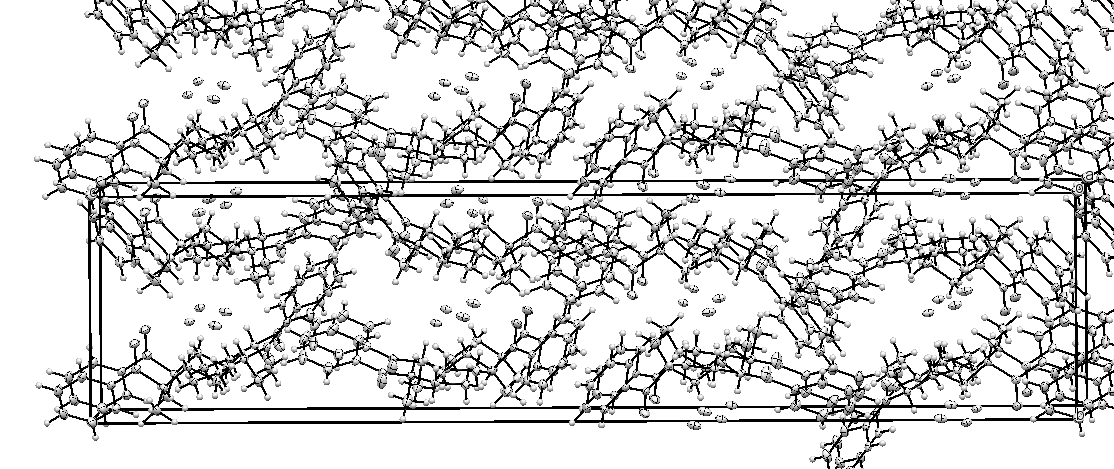


**Fig. S12** The packing along a axis of compound **1**


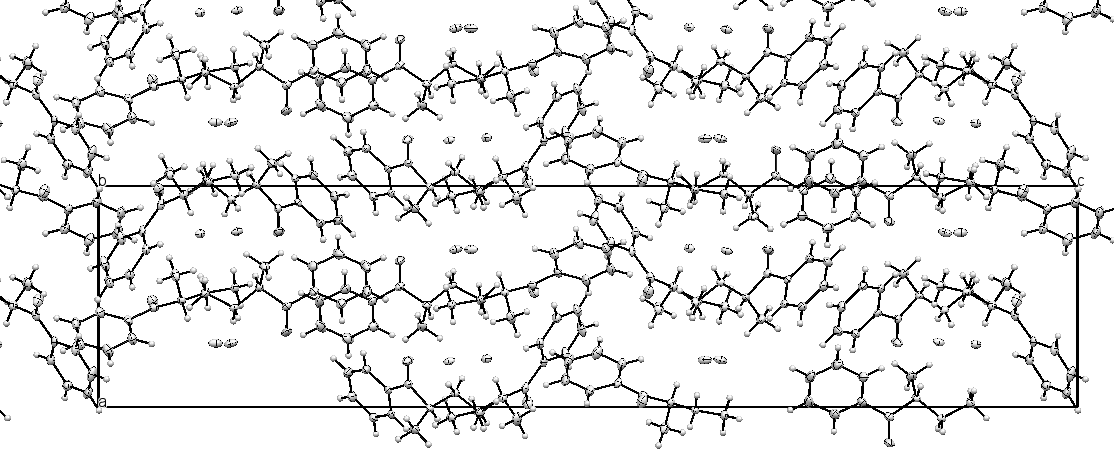


**Fig. S13** The packing along b axis of compound **1**

**
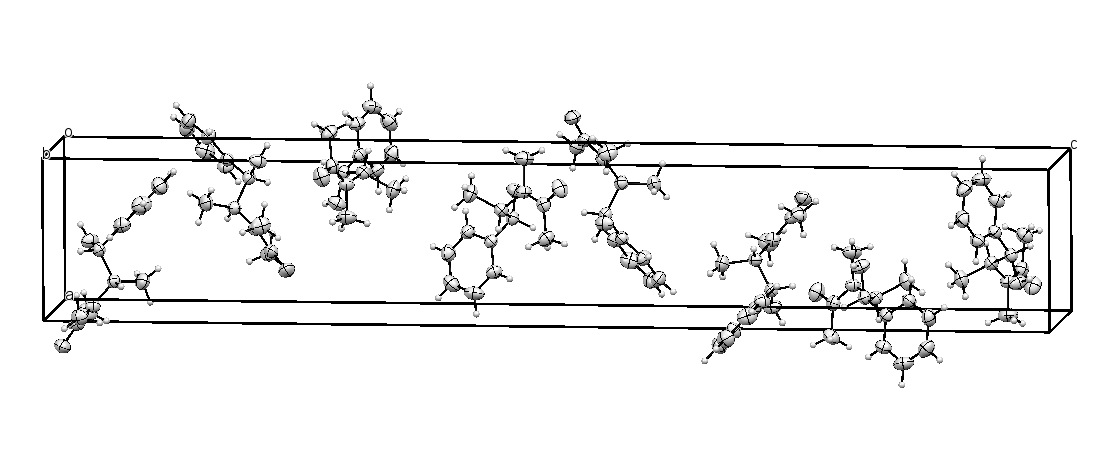
**

**Fig. S14** Packing diagram of **2**. View along b axis

**
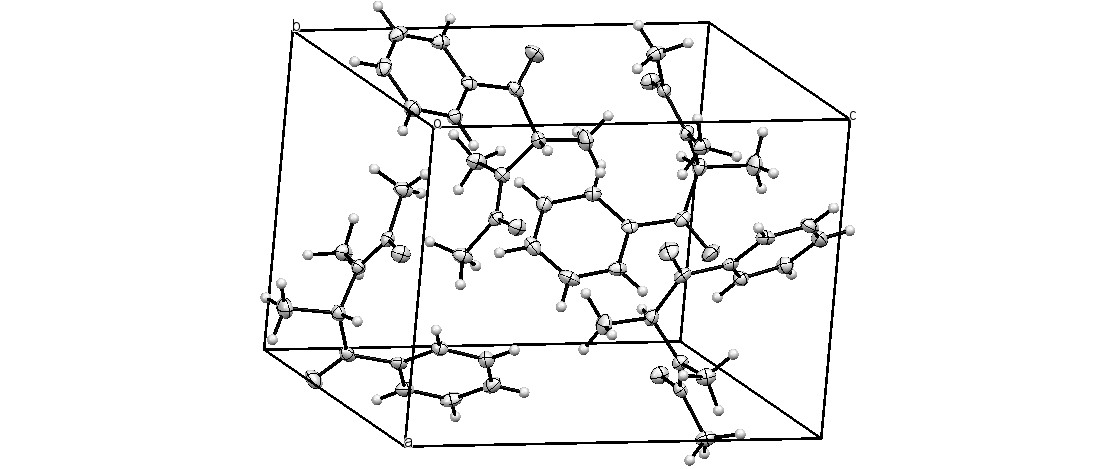
**

**Fig. S15** Packing diagram of **3**. View along b axis


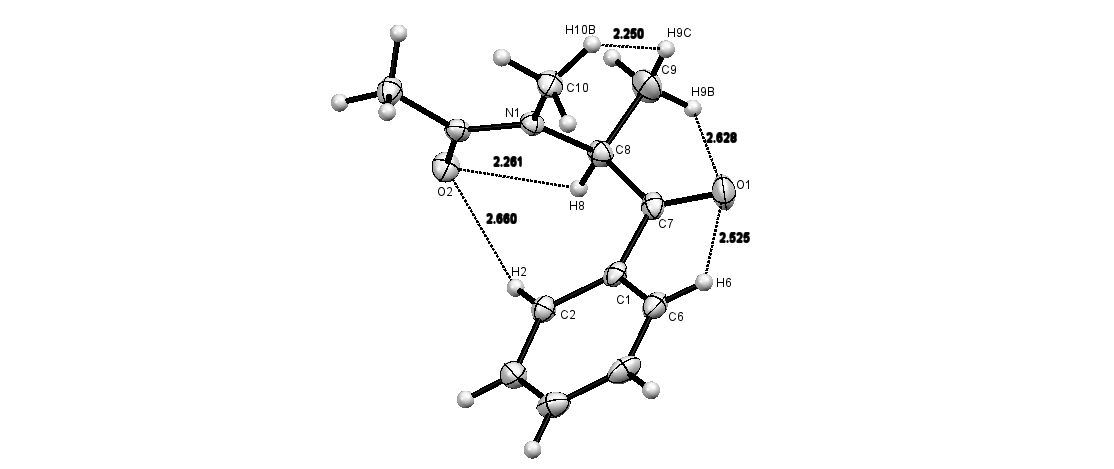


**Fig. S16** Weak hydrogen bonds occurring between fragments of compound **3**


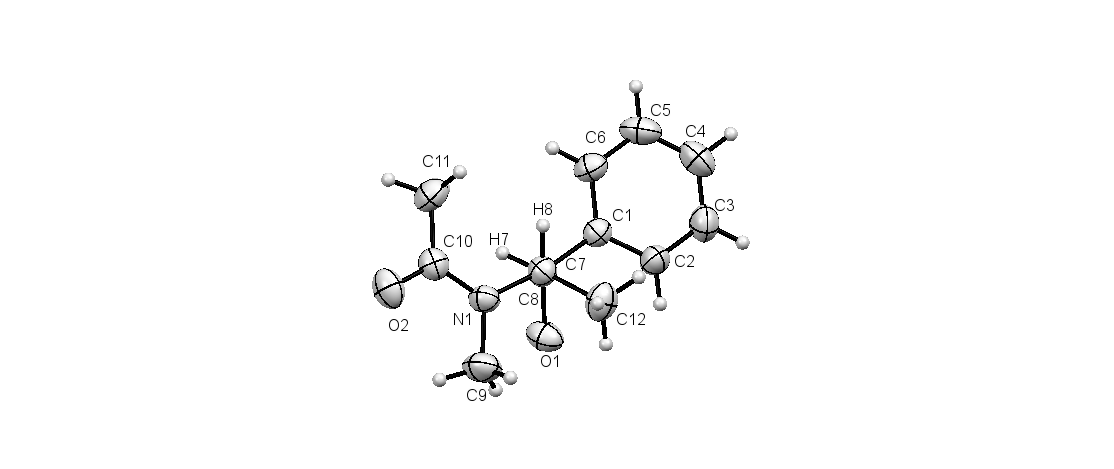


**Fig. S17** Scheme and actual structure of conformer **2a** occurring in crystals of compound **2**


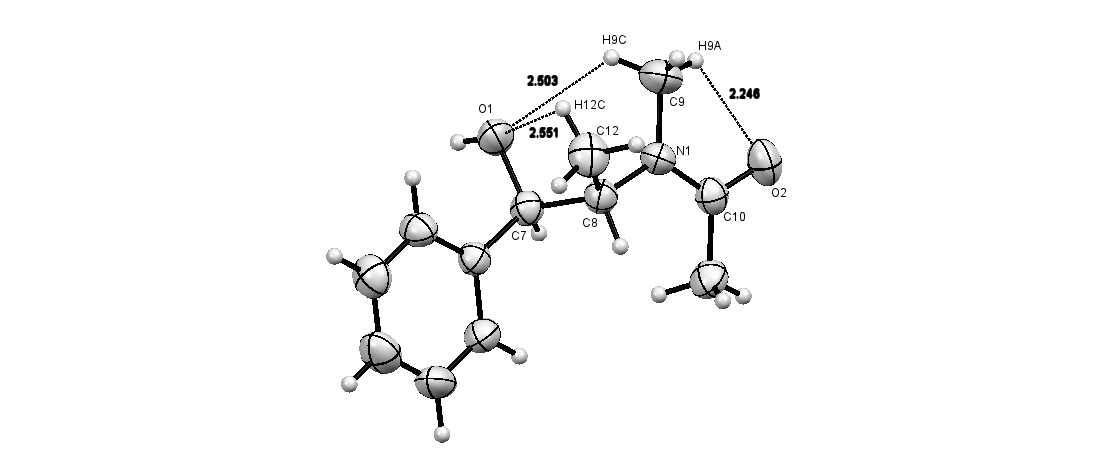


**Fig. S18** Weak hydrogen bonds keeping compound **2** molecules in the alternate conformation
